# Very Low Food Security Among Low-Income Households With Children in California Before and Shortly After the Economic Downturn From COVID-19

**DOI:** 10.5888/pcd18.200517

**Published:** 2021-01-07

**Authors:** Fred Molitor, Celeste Doerr

**Affiliations:** 1 California State University Sacramento, Department of Communication Studies, Sacramento, California; 2 Public Health Institute, Center for Wellness and Nutrition, Sacramento, California

## Abstract

We examined levels of very low food security (VLFS) among low-income households with children in California before and shortly after the economic downturn from coronavirus disease 2019 (COVID-19). Households were randomly sampled in 2018, 2019, and 2020; 11,653 mothers were administered the US Department of Agriculture 6-item Food Security Survey Module. Post–COVID-19 (April 27 to July 21, 2020, a period when stay-at-home restrictions were eased in the state), 14.0% of mothers reported VLFS versus 19.3% pre–COVID-19 (November 21, 2019, to March 14, 2020) (*P* = .003), 22.2% in 2019 (*P* < .001), and 19.0% in 2018 (*P* = .004). Existing systems to quickly obtain food assistance benefits in California and new federal benefits available in response to COVID-19 may have reduced VLFS.

**Summary**

**What is already known on this topic?**

Since 2018, annual population-based surveys have found approximately 20% of low-income families in California report very low food security.

**What is added by this report?**

The Families First Coronavirus Response Act and the Coronavirus Aid, Relief, and Economic Security Act allowed California to increase Supplemental Nutrition Assistance Program benefits and distribute 2.9 million Pandemic Electronic Benefit Transfer cards to purchase food. Concurrently, during April through July 2020, very low food security among low-income households with children decrease to 14%.

**What are the implications for public health practice?**

Enhanced safety-net benefits coupled with responsive systems can rapidly decrease food insecurity among at-risk populations.

## Objective

The California Family Health Study (CFHS) is an annual population-based telephone survey of mothers from low-income households occurring throughout each federal fiscal year (FFY, October 1 through September 30). Since 2018, the CFHS has included the US Department of Agriculture (USDA) 6-item Food Security Survey Module (1). On March 16, 2020, survey operations ceased as nonessential businesses in California were closed in response to coronavirus disease 2019 (COVID-19) (2). From March to May the seasonally adjusted unemployment rate in California increased from 5.3% to 16.3% (3). Sixty-four percent of lost jobs were in low-paying industries (4). African American women and Latina women were twice as likely to lose their jobs as White women, and immigrants were more likely to become unemployed than nonimmigrants (4). CFHS interviews resumed on April 27, 2020, as stay-at-home restrictions were eased, but interviews were terminated 3 months later, again because of COVID-19.

During the March to April CFHS shutdown, the Families First Coronavirus Response Act (FFCRA) and the Coronavirus Aid, Relief, and Economic Security (CARES) Act were signed into law. In California, Supplemental Nutrition Assistance Program (SNAP) benefits were increased and Pandemic Electronic Benefit Transfer (P-EBT) cards to purchase food were distributed. Our objective was to examine the increase in publicly funded economic assistance programs in relation to food insecurity among low-income California families before and shortly after the economic downturn from COVID-19.

## Methods

Verbal consent was documented for all mothers participating in the 2018 through 2020 CFHS. The California Health and Human Services Agency, Committee for the Protection of Human Subjects, approved the study protocols.

Households at or below 185% of the federal poverty level with 1 or more adult woman and child(ren) 5 to 17 years were sampled at random. CFHS procedures include removing from the annual sampling frames any households that were previously recruited. Recruitment involved a letter of introduction to the study, in English or Spanish, followed by a telephone call from bilingual staff to verify household eligibility and identify the youngest mother (or caregiver) in the household. A $15 gift card was offered. Telephone interviews were scheduled with mothers who expressed interest.

Race/ethnicity was assessed by asking, “Are you Hispanic, Latina, or of Spanish origin?” and “What is your race? You may answer more than one. Are you American Indian or Alaska Native, Asian, Black or African American, Native Hawaiian or other Pacific Islander, White, or other?” Age and highest level of education were also recorded.

Mothers providing affirmative responses to at least 5 of the 6 items of the Food Security Survey Module were assigned the status of very low food security (VLFS), in accordance with established procedures (1). Mothers were also coded for the time period of the interview, in FFY 2018, FFY 2019, 2020 pre–COVID-19 (November 21, 2019, to March 14, 2020), or 2020 post–COVID-19 (April 27 to July 21, 2020).

One logistic regression model examined the proportion of VLFS households across the 4 time periods (reference group, 2020 post–COVID-19). Covariates were age (centered on the mean); highest level of education (reference group was less than high school graduate compared with high school graduate and some college or higher); and race/ethnicity (reference group was Latina compared with African American, White, and other or missing).

Excluded from the analyses were records missing all Food Security Survey Module items (146 in 2018, 230 in 2019, and 10 in 2020). Data merging, cleaning, coding, and analyses were conducted with SPSS (version 26.0, IBM Corp).

## Results

Valid data were recorded from 11,653 mothers. The majority (65.7%) were Latina, 12.6% were African American, and 16.8% were White.

In 2020 post–COVID-19, 14.0% of mothers reported VLFS. In 2020 pre–COVID-19, VLFS was 19.3% (adjusted odds ratio [aOR], 1.49; *P* = .003). In 2019 and 2018, VLFS was 22.2% (aOR, 1.77; *P* < .001) and 19.0% (aOR, 1.47; *P* = .004), respectively (Table).

**Table:** Logistic Regression Models of Very Low Food Security Reported by Mothers From Low-Income Households, California Family Health Study, 2018–2020

Survey Year	No./Total Surveyed (%)	Odds Ratio (95% CI)	Adjusted Odds Ratio^a^ (95% CI)
2020 Post–COVID-19^b^	81/579 (14.0)	1 [Reference]	1 [Reference]
2020 Pre–COVID-19^c^	341/1,765 (19.3)	1.47 (1.13–1.92)	1.49 (1.15–1.94)
2019^d^	1,195/5,384 (22.2)	1.75 (1.38–2.24)	1.77 (1.39–2.26)
2018^e^	747/3,925 (19.0)	1.45 (1.13–1.85)	1.47 (1.14–1.89)

a Covariates were race/ethnicity, age, and highest level of education.

b Interviews conducted from April 27 to July 21, 2020, a period when stay-at-home restrictions were eased in the state.

c Interviews conducted from November 21, 2019, to March 14, 2020.

d The federal fiscal year was used, October 1, 2018, through September 30, 2019.

e The federal fiscal year was used, October 1, 2017, through September 30, 2018.

To test whether seasonality may explain these findings, VLFS rates were compared by using the 2020 time periods in 2018 and in 2019. In 2018, VLFS was 1.7 percentage points higher in the April to July period compared with the November to March period (*P* = .31); in 2019, VLFS was 3.1 percentage points lower in the April to July period compared with the November to March period (*P* = .06 both by χ^2^ test).

## Discussion

The 11-percentage-point upsurge in unemployment following California’s executive order to stay at home in response to COVID-19 (3) coincided with a significant decrease in VLFS among low-income households with children.

Existing systems to quickly identify and enroll eligible families in SNAP (CalFresh in California) and responsive actions to the COVID-19 pandemic by the federal government and California Department of Social Services (CDSS) may explain these findings. In 5 months (February to June 2020), California households receiving CalFresh benefits increased 21%, or by 463,725 households (5). Nord and Golla documented rates of VLFS decreasing from 20% to 12% within 2 months from SNAP enrollment (6), well within the time frame for 2020 CFHS-eligible mothers to have benefited from CalFresh. The FFCRA and CARES Act (both signed into law in March 2020) facilitated increased access to resources to purchase food among low-income families in California in 2 ways. First, the CDSS raised CalFresh-eligible households’ allotment to the maximum allowable based on household size (7). These emergency funds were available on April 12, 2020, two weeks before 2020 CHFS interviews resumed. Second, P-EBT cards, providing families up to $365 per eligible child, were distributed beginning on May 11, 2020 (8). A total of 2.9 million P-EBT cards were distributed, reaching 93% of children from eligible families, with $986 million redeemed to purchase food (Brian Kaiser, Bureau Chief, CalFresh and Nutrition Programs, CDSS, email, August 11, 2020). Similar to the findings reported by Brown and Tarasuk following the implementation of the Canada Child Benefit program (9), our study’s findings suggest that publicly funded economic assistance programs can decrease food insecurity.

Our findings are subject to self-report biases. However, it is reasonable to assume that any related biases would be stable over time in a population-based sample. Changes in the receipt of CalFresh benefits among CFHS participants would assist in interpreting the findings but were not included as CFHS survey items. Study strengths include random selection of households across California and standardized recruitment and interview procedures.

Rates for VLFS among low-income families in California, as documented since 2018, dropped by 5 percentage points within 1 to 4 months following COVID-19 stay-at-home restrictions. Possible explanations include ongoing and enhanced COVID-19–specific CalFresh benefits. Whether federal and California-specific safety-net benefits will have a lasting effect on levels of VLFS and, in turn, chronic diseases associated with food insecurity (10,11), requires ongoing study.
